# Cysteamine-mediated clearance of antibiotic-resistant pathogens in human cystic fibrosis macrophages

**DOI:** 10.1371/journal.pone.0186169

**Published:** 2017-10-05

**Authors:** Chandra L. Shrestha, Kaivon D. Assani, Hannah Rinehardt, Florentina Albastroiu, Shuzhong Zhang, Richard Shell, Amal O. Amer, Larry S. Schlesinger, Benjamin T. Kopp

**Affiliations:** 1 Center for Microbial Pathogenesis, The Research Institute at Nationwide Children’s Hospital, Columbus, Ohio, United States of America; 2 Section of Pediatric Pulmonology, Nationwide Children’s Hospital, Columbus, Ohio, United States of America; 3 Department of Microbial Infection and Immunity, The Ohio State University, Columbus, Ohio, United States of America; 4 Pulmonary, Allergy, Critical Care and Sleep Medicine, Davis Heart and Lung Research Institute, Department of Internal Medicine, The Ohio State University, Columbus, Ohio, United States of America; Louisiana State University, UNITED STATES

## Abstract

Members of the *Burkholderia cepacia* complex are virulent, multi-drug resistant pathogens that survive and replicate intracellularly in patients with cystic fibrosis (CF). We have discovered that *B*. *cenocepacia* cannot be cleared from CF macrophages due to defective autophagy, causing continued systemic inflammation and infection. Defective autophagy in CF is mediated through constitutive reactive oxygen species (ROS) activation of transglutaminase-2 (TG2), which causes the sequestration (accumulation) of essential autophagy initiating proteins. Cysteamine is a TG2 inhibitor and proteostasis regulator with the potential to restore autophagy. Therefore, we sought to examine the impact of cysteamine on CF macrophage autophagy and bacterial killing. Human peripheral blood monocyte-derived macrophages (MDMs) and alveolar macrophages were isolated from CF and non-CF donors. Macrophages were infected with clinical isolates of relevant CF pathogens. Cysteamine caused direct bacterial growth killing of live *B*. *cenocepacia*, *B*. *multivorans*, *P*. *aeruginosa* and MRSA in the absence of cells. Additionally, *B*. *cenocepacia*, *B*. *multivorans*, *and P*. *aeruginosa* invasion were significantly decreased in CF MDMs treated with cysteamine. Finally, cysteamine decreased TG2, p62, and beclin-1 accumulation in CF, leading to increased *Burkholderia* uptake into autophagosomes, increased macrophage CFTR expression, and decreased ROS and IL-1β production. Cysteamine has direct anti-bacterial growth killing and improves human CF macrophage autophagy resulting in increased macrophage-mediated bacterial clearance, decreased inflammation, and reduced constitutive ROS production. Thus, cysteamine may be an effective adjunct to antibiotic regimens in CF.

## Introduction

Cystic fibrosis (CF) is characterized by chronic sinopulmonary bacterial and fungal infections leading to inflammation, tissue damage, and accelerated loss of lung function [1]. Emerging evidence suggests that failure to clear bacterial infections in CF is in part due to impaired host immune defenses [2–5]. Deficiencies in innate immune responses combined with extensive bacterial biofilm formation [6, 7] may also explain why many chronic bacterial infections in CF are recalcitrant to antibiotic treatment, promoting their persistence in CF airways.

One mechanism by which pathogens can subvert host immune responses to increase their survival in CF is through altering macrophage autophagy [2, 8, 9]. Autophagy is a physiologic process whereby cellular components and/or pathogens can be packaged into autophagosomes for degradation [10]. Autophagy regulation in CF is mediated through a complex process, and found to be decreased at baseline [11]. Neutrophil-driven over-production of reactive oxygen species (ROS) in CF airways due to failed bacterial clearance leads to ROS-mediated activation of the enzyme Transglutaminase-2 (TG2). TG2 subsequently leads to cross-linking of beclin-1 into aggresomes, thereby favoring the sequestration of essential autophagy initiating molecules [11–13]. Restoration of functional autophagy reduces inflammation [11] and improves bacterial killing in CF [2], but it is unknown how TG2 specifically mediates human macrophage bacterial killing.

Members of the *Burkholderia cepacia* complex are virulent, multi-drug resistant pathogens that survive and replicate intracellularly in patients with CF. We have discovered that *B*. *cenocepacia* cannot be cleared from CF macrophages due to defective autophagy, causing continued systemic inflammation and infection. [2, 9, 14] *B*. *cenocepacia* can further suppress autophagy independent of the host [2, 15], but its connection to TG2 is unclear. Autophagy is integral in the clearance of other common CF pathogens [16, 17]. Therefore, we sought to examine the impact of cysteamine, a TG2 inhibitor, on human CF macrophage autophagy and bacterial killing. Cysteamine is an aminothiol degradation product of the amino acid cysteine, and is currently FDA approved for nephropathic cystinosis in an available oral formulation [18]. Cysteamine has mucolytic, anti-biofilm, and bactericidal properties in pre-clinical CF studies [19–21], and has demonstrated lung bioavailability in early CF clinical trials [22]. Cysteamine also increases autophagy in CF epithelial cells [23]. We hypothesized that cysteamine would decrease macrophage TG2 production leading to improved autophagy, decreased bacterial load, and alleviated inflammation.

## Materials and methods

### Ethics statement

All human subjects were recruited as approved by the Institutional Review Board of Nationwide Children’s Hospital. All subjects underwent written consent for the procedures including all adult subjects provided informed consent, and a parent or guardian of any child participant provided informed consent on their behalf along with written assent from children.

### Bacterial strains and culture

Macrophages were infected with RFP-expressing *B*. *cenocepacia* strain k56-2, *B*. *multivorans* strain FC-445, and CF clinical isolates of *Pseudomonas aeruginosa* and methicillin-resistant Staphylococcus aureus (MRSA) [2, 14, 24], all grown for 24h in LB media. The *B*. *cenocepacia* strain is representative of an epidemic clinical strain from the ET12 lineage [25]. For intracellular infection studies a gentamicin-sensitive strain of *B*. *cenocepacia* (MH1K) was used in place of k56-2. Enteric strains used in the bacterial killing assay were grown for 24h and included *Escherichia coli* K12 (aerobic conditions, no C02, Fisher BioReagents^™^ Microbiology Media: LB Broth, Miller), *Salmonella typhimurium* JSG 210 (LB Broth, aerobic, no CO2), Citrobacter rodentium DBS210 (LB broth, no CO2), *Porphyromonas gingivalis* ATCC 33277m (LB broth, no CO2, no shaking), *Bifidobacterium animalis* Align (LB broth, CO2, no shaking), and *Lactobacillus reuteri* ATCC 23272 (MRS broth, Difco Laboratories). Enteric strains kindly donated by Michael Bailey, NCH.

### Macrophage isolation

Heparinized blood was obtained from CF and non-CF healthy controls. Subjects were excluded with a history of chronic immunosuppression including chronic steroid use, or history of transplantation. Peripheral monocytes were separated from whole blood using Lymphocyte Separation Medium (Corning, 25-072-CV) and differentiated for 5 days at 37°C into monocyte-derived macrophages (MDMs) as previously described [2, 14, 26]. MDMs were cultivated in non-stick Teflon wells with limited adherence to facilitate removal prior to attachment in tissue culture plates. Isolated MDMs were washed by centrifugation and confirmed microscopically and by flow cytometry, placed in monolayer culture, and infected according to experiments. Alveolar macrophages were isolated from human bronchoalveolar lavage fluid after centrifugation and re-suspension in RPMI and confirmed by flow cytometry. Macrophages were attached to plates for 24h prior to experimentation.

THP-1 cells were grown in 10% fetal bovine serum (FBS, Thermo scientific) in RPMI. THP-1 cells were treated with 200nM PMA (Calbiochem, 524400) and 20ng/mL GM-CSF (R&D Systems, 415-ML-050) for 24h to differentiate cells into macrophages and then replenished with RPMI plus FBS. Media was replenished with 20ng/mL GM-CSF the next day and the THP-1 derived macrophages were allowed to mature 5 days before experimentation. THP-1 cells were then treated with the cystic fibrosis transmembrane conductance regulator (CFTR) inhibitor Inh-172 (Sigma, C2992) for 24 h prior to experimentation. THP-1 derived macrophages were used in preliminary experiments prior to confirmation and further experimentation with MDMs or alveolar macrophages.

### Macrophage infection studies

For colony forming unit (CFU) analysis, 0.5x10^6^ macrophages were plated in 24 well plates (Corning Inc. 353047). Cysteamine was added one day prior to infection. Subsequently, MDMs were infected with bacteria at an MOI of 10 and incubated for 1h at 3°C and 5% C02. After 1h the macrophages were washed with RPMI and then complete media containing 10% human AB serum in RPMI was added. For cysteamine washout experiments, after 24h of cysteamine exposure macrophages were washed with RPMI and replenished with complete media to remove cysteamine. Macrophages were then rested overnight prior to infection. For intracellular CFU assays, *B*. *cenocepacia*, *B*. *multivorans*, and *P*. *aeruginosa* wells were treated with gentamicin (50 μg/ml gentamicin, Invitrogen 3564), and MRSA wells were treated with linezolid (40 μg/ml, Sigma PZ0014) for 2h to kill extracellular bacteria. For total CFU assays, no antibiotics were added. For enumeration of intracellular bacteria the macrophages were lysed with PBS solution containing 0.1% Triton X-100 (Acros Organics 9002-93-1) for 10 min and cells were scraped using the plunger from a BD 1mL TB Syringe pipetted up and down as described [27]. Recovered bacteria were quantified by plating serial dilutions on LB agar plates and analyzed for CFUs. For immunoblotting studies, MDMs were infected at a MOI of 10 for 1 or 24h depending on experimental conditions. For oxidative burst studies, MDMS were infected at a MOI of 10 for 1h prior to measuring ROS production. For TEM, MDMs were isolated and infected with *B*. *cenocepacia* at an MOI of 10 for 1h prior to experimentation. For confocal microscopy, MDMs were infected synchronously with bacteria at an MOI of 2 for ease of counting (an MOI of 10 renders counting of bacteria in untreated CF MDMs difficult due to clumping). For ELISA, MDMs were infected at an MOI of 10 for 1h prior to measuring cytokine production over 24h.

### Macrophage apoptosis

MDMs were plated at a density of 3x 10^6^/ml in 12-well plates (BD Falcon, 353043). Cysteamine was added 24h before infection. The cells were infected with *B*. *cenocepacia* at an MOI of 10. Viability assay was done by FACS analysis using the APC Annexin V apoptosis detection kit with Propidium Iodide (PI) (Biolegend 640932). The macrophages were detached by Accutase cell detachment solution (Corning 25058C1). Cells were collected, washed, and re-suspended in 100 μl of Annexin V Binding Buffer (Biolegend 422201), and then stained with 3 μl of Annexin V and 5 μl PI in the dark for 20 min at room temperature. The percentage of non-viable, apoptotic cells was assessed using flow cytometry (BD LSR 11 Flow Cytometer; BD Bioscience).

### Direct bacterial growth

Bacteria were grown for 24h and 4x10^8^ CFUs of bacteria were added in 200 μl of LB broth in 96-well plates with or without desired treatments. The control wells contained LB liquid media only. Serial dilution was performed up to 10^−7^ and cysteamine (Sigma, M9768) was added at a concentration of 10mM at the start or 3 h after incubation with bacteria as noted. For bacterial growth, serial dilutions were plated in LB agar plates and incubated for 24h at 37°C and CFUs were counted. For bacterial inhibition assays, 600nm optical wavelengths were measured on a Synergy H1 Hybrid Reader spectrophotometer (Biotech, Biotech Instruments, Vermont, USA) over 24h with shaking at every 15 min at 37°C.

### Immunoblotting

Macrophages were plated at a density of 4x10e6/ml in a 12-well plate (BD Falcon, 353043). Cysteamine was added for 24h prior to infection and during infection. Supernatants were removed post experimentation and the cells were washed twice with phosphate buffered saline (PBS, Fisher Scientific). The cells were lysed in lysis buffer (HEPES, MgCl_2_, EGTA, KCl, NP-40) with protease inhibitor (Roche Applied Science, 10-519-978-001) and use of a cell scraper (Sarstedt, 83–1832). After collecting all the contents, the sample was centrifuged at 14,000 RPM for 10 min at 4°C. Then, the supernatant was placed on ice. Total protein content was determined by Bradford Assay using the Bradford Reagent (Bio- Rad, 500–0207) and Standards (Bio-Rad, 500–0207). Five μl of sample or standard were loaded in duplicates on a 96 well flat bottom plate (Corning, 3370) and 250 μl of Bradford Reagent was added to each well. The samples were incubated for 5–10 min at room temperature and absorbance was measured at 595 nm on a Synergy H1 Hybrid Reader spectrophotometer (Biotech, Biotech Instruments, Vermont, USA). The quantification of total protein of each sample was determined by plotting a standard curve of BSA standard and determined the concentration of samples. Then, 30 μg of protein was separated by SDS-PAGE using Bolt Bis-Tris Plus gels (Invitrogen NW04122 BOX). After gel separation, the protein was transferred to an IBlot 2 PVDF Transfer Stack (Invitrogen IB24001) using an IBlot 2 gel Horizontal Transfer Device (Invitrogen IB21001) for 7 mins. Membranes were immunoblotted for calreticulin (Enzo Life Sciences, ADI-SPA-600-F), LC3 (Sigma, L8918- 200), p62 (Sigma, P0067-200), beclin-1 (Abcam, ab55878), CFTR (R&D Systems, MAB 25031), TG2 (Santacruz- Cat #- sc20621), and IL-1β (kindly provided by Mark Wewers, OSU). Protein bands were detected with HRP-conjugated secondary antibodies and visualized using enhanced chemiluminescence (ECL) reagents (GE Healthcare, RPN2106).

### DCF assay

The oxidative burst was measured by a 2′,7′-dichlorofluorescein (DCF) assay, (Life Technologies, D399) using relative fluorescent units (RFUs). MDMs were adhered to 96 well plates at 0.8e6 cells/well in duplicate for 2h, and then cysteamine added for 24h prior to infection and during infection. Then cells were repleted in Dulbecco’s PBS + 10mM HEPES + 1 mg/ml human serum albumin, CaCl_2_ + 0.1% glucose (DPBS-HHG) in each well for the DCF assay only. After 30 min incubation at 37°C, 10%DCF was added to the wells for 30 min at 37°C. MDMs were then infected and DCF fluorescence was measured at a 485nm excitation wavelength and a 515nm emission wavelength every 2 min for 2 h.

### Transmission electron microscopy

TEM Images were obtained using a FEI Technai G2 Spirit transmission electron microscope (FEI, USA), Macrofire (Optronics) digital camera and AMT image capture Software with assistance from the Campus Microscopy and Imaging Facility (CMIF) at The Ohio State University. Cells were cultured on Permanox (Lab-Tek) chamber slides and fixed with 2.5% gluteraldehyde in 0.1M phosphate buffer with 0.1M sucrose. Slides were post fixed with 1% osmium tetroxide in phosphate buffer then en bloc stained with 2% uranyl acetate in 10% ethanol, dehydrated in a graded series of ethanols and embedded in Eponate 12 epoxy resin (Ted Pella Inc., USA). Ultrathin sections were cut on a Leica EM UC6 ultra microtome (Leica microsystems, Germany), collected on copper grids, and then stained with lead citrate and uranyl acetate.

### Confocal microscopy

One million MDMs were cultured on 22 mm glass cover slips in 24-well tissue culture plates aseptically overnight. Cells were then washed with warm RPMI 3 times and replenished with RPMI (Corning, 10041CV) and 10% Human AB serum (Corning, 35-060-Cl). Cysteamine was added 24h before infection and during infection. MDMs were infected with *B*. *cenocepacia* at an MOI of 2 for 24h. The media was then aspirated and 200 μl of 4% paraformaldehyde (PFA) were added to the mixture (Affymetrix 19943) and incubated for 30 min at room temperature in the dark. The PFA was aspirated, cells washed 3 times with PBS and then 200 μl of cold methanol was added for 10 seconds. Next, 500 μl of Blocking Buffer [5% cold heat-inactivated (HI) goat serum in PBS] was added in each well and cells incubated for 1h at 37°C. Two hundred μl of primary antibody LC3 (Abgent, AP1805a, 1:100), Beclin-1 (Abcam, ab55878, 1:100), or P62 (Sigma, P0067-200, 1:100) were added in wells and with incubation for 1h at 37°C. Antibodies were aspirated and cells washed 3 times in blocking buffer. Two hundred μl of secondary antibody Alexa Flour 488 goat anti-rabbit (Invitrogen Molecular probes, A11008, 1:5000) were added and wells incubated for 1h at 37°C. Subsequently, the secondary antibody was aspirated and 200 μl of 4',6-diamidino-2-phenylindole (DAPI, 1:5000) were added for 5 min at room temperature and cells washed 3 times with blocking buffer. The coverslip was mounted on the slide with Prolong Gold anti-fade reagent with DAPI (Molecular Probes Life Technologies, REF P36935). Confocal microscopy was performed using an Axiovert 200M inverted epifluorescence microscope equipped with the Apotome attachment for improved fluorescence resolution and an Axiocam MRM CCD camera (Carl Zeiss Inc., Thornwood, NY). At least 100 macrophages were scored for each condition. All experiments were performed in triplicate. Quantification of beclin-1 density was performed with ImageJ software using mean area of pixel density of beclin-1 fluorescence.

### Enzyme-linked immunosorbent assay (ELISA)

MDM culture supernatants were isolated, filtered, and stored at -80°C after 24h infection with *B*. *cenocepacia* and cysteamine. IL-1β quantification was determined by sandwich ELISA following the manufacturer’s protocol (R&D system Inc., DY201) as previously described [28].

### Statistical analysis

Statistical analyses were performed using GraphPad Prism software (version 6.1). Two sample t-tests or Mann-Whitney U tests were used for independent sample comparisons. One-way ANOVA with Tukey correction was performed for multiple comparisons. Statistical significance was defined as a p value <0.05. Age and gender matched healthy controls were used for comparison.

## Results

### Cysteamine increases bacterial killing

**Fig 1:**
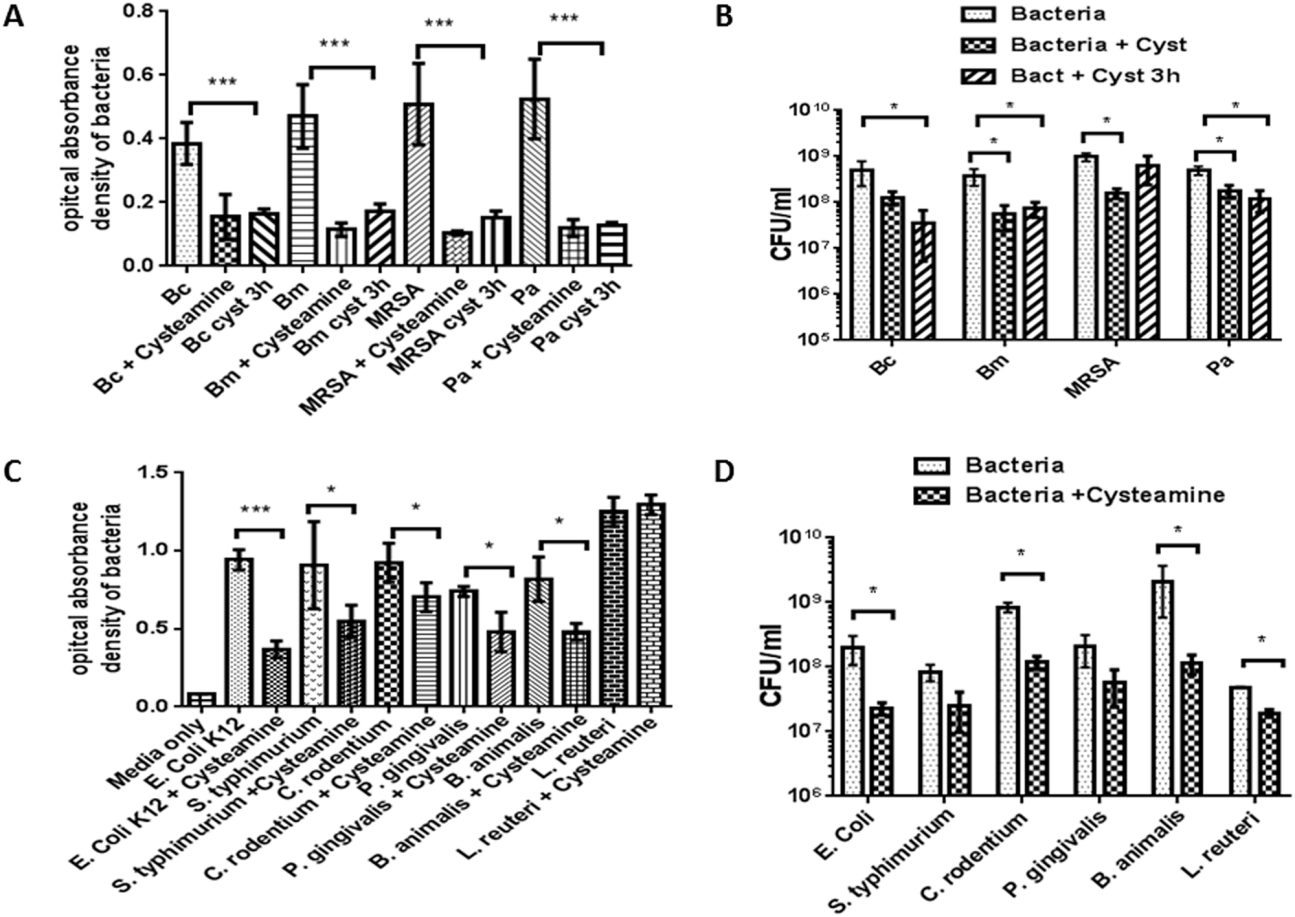
Cysteamine increases bacterial killing. A) Summed end-point analysis of 24h bacterial killing assay of cysteamine against multi-drug resistant *B*. *cenocepacia* (Bc), *P*. *aeruginosa* (Pa), Methicillin-resistant *Staphylococcus aureus* (MRSA), and *B*. *multivorans* (Bm) in media devoid of human cells. NT = media alone, 3h = cysteamine added 3h after the start of culture, n = 3. B) Colony-forming units (CFU) for bacteria during 1A conditions, n = 4. C) Summed end-point analysis of 24h direct bacterial killing assays of cysteamine against enteric pathogens in media: *Escherichia coli* (*E*. *coli*), *Salmonella typhimurium* (*S*. *typhimurium*), *Citrobacter rodentium* (*C*. *rodentium*), *Porphyromonas gingivalis* (*P*. *gingivalis*), *Bifidobacterium animalis* (*B*. *animalis*), and *Lactobacillus reuteri* (*L*. *reuteri*), n = 3. “*” denotes a p value < 0.05, “**” denotes a p value < 0.01, and “***” denotes a p value < 0.001 by ANOVA for 1A and t-test for 1B, 1C. Means and standard deviation reported. D) Colony-forming units (CFU) for bacteria during 1C conditions, n = 4.

Cysteamine has direct antimicrobial properties against common CF pathogens with a range of minimal inhibitory concentrations (MIC) [19, 20]. We sought to extend this finding using antibiotic-resistant clinical isolates of 4 common CF pathogens. Direct bacterial killing was determined in media containing cysteamine. Cysteamine demonstrated significant direct bacterial killing of *B*. *cenocepacia*, *B*. *multivorans*, *P*. *aeruginosa*, and MRSA when added at the beginning of the culture (Fig 1A). Additionally, cysteamine was able to increase bacterial killing for all 4 pathogens when added after 3 hours of culture during the log phase of bacterial growth (Fig 1A). We then measured bacterial CFUs in the media and found that cysteamine reduced *B*. *cenocepacia*, *B*. *multivorans*, *P*. *aeruginosa*, and MRSA CFU by approximately one log (Fig 1B). The addition of cysteamine at 3 hours of culture significantly increased bacterial killing for all pathogens except for MRSA, which did not manifest reduced CFUs under this condition. Due to the broad antimicrobial properties of cysteamine and known gastrointestinal side effects [22], we next tested for direct bacterial killing against common enteric pathogens to determine if cysteamine’s impact was specific to CF pathogens or more general in nature. Cysteamine demonstrated significant bacterial killing against *E*. *coli*, *S*. *typhimurium*, *C*. *rodentium*, *P*. *gingivalis*, and *B*. *animalis*, but not against *L*. *reuteri* (Fig 1C). However, significant reductions in end-assay bacterial CFUs were demonstrated only for *E*. *coli*, *C*. *rodentium*, *B*. *animalis*, and *L*. *reuteri* (Fig 1D).

### Cysteamine increases bacterial killing in CF macrophages

**Fig 2:**
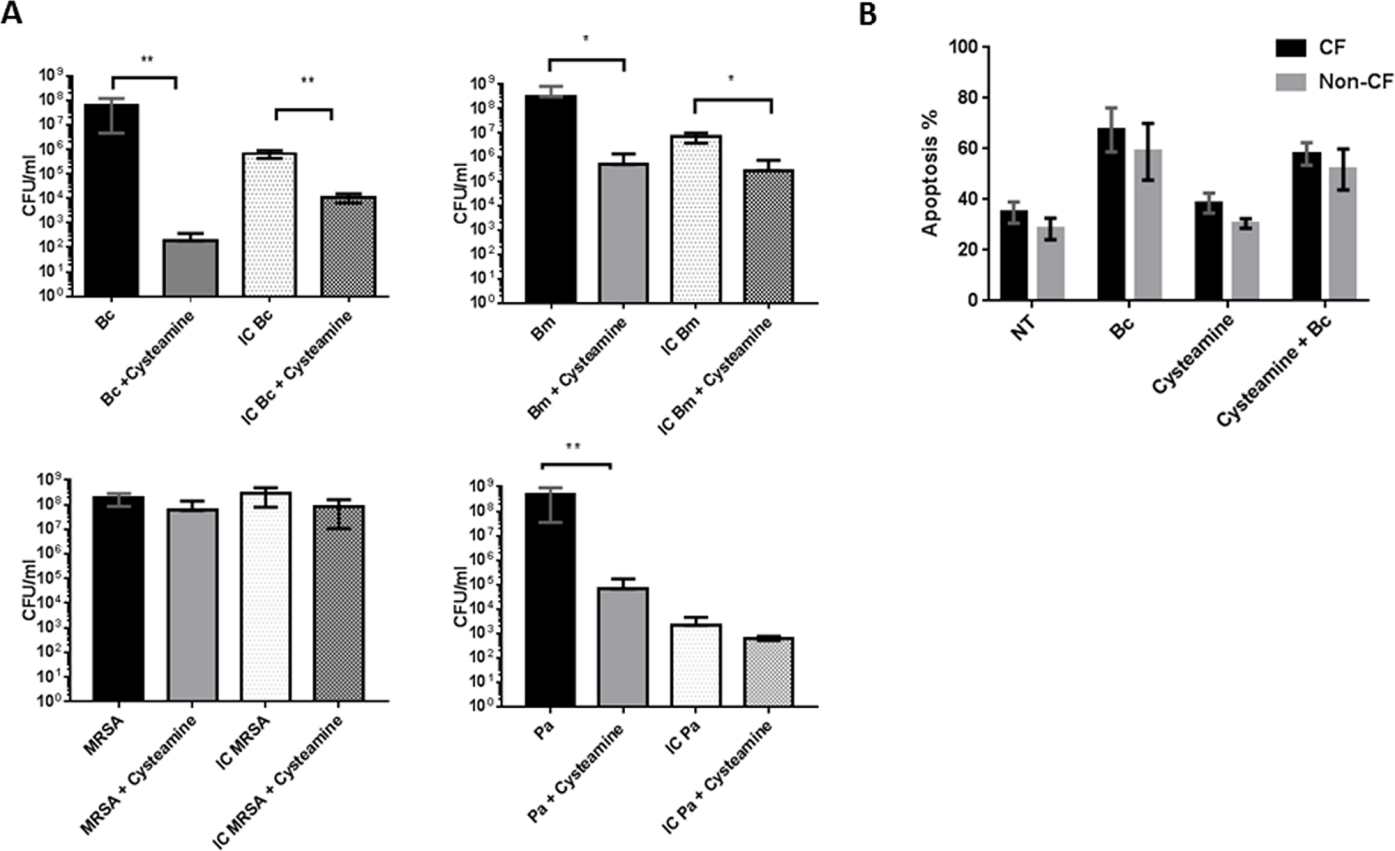
Cysteamine decreases bacterial loads in CF macrophages. A) Colony forming unit (CFU) assay for human CF MDMs infected with *B*. *cenocepacia* (Bc), *B*. *multivorans* (Bm), *P*. *aeruginosa* (Pa), and MRSA for 24h with or without cysteamine, n = 3. Total and intracellular (IC) only counts are displayed. “*” denotes a p value < 0.05. “**” denotes a p value < 0.01. B) Macrophage apoptosis assay for human CF and non-CF MDMs uninfected (NT), during *B*. *cenocepacia* infection (Bc), exposure to cysteamine, and during *B*. *cenocepacia* infection and combined cysteamine exposure. Results are presented as % non-viable macrophage apoptosis as measured by flow cytometry, n = 4.

We have shown that highly virulent bacteria such as *B*. *cenocepacia* can avoid normal host defenses by surviving intracellularly in human CF macrophages [2, 14]. Recently, cysteamine was found to improve the clearance of *P*. *aeruginosa* from murine CF F508del macrophages [21]. The ability of these pathogens to replicate in human host cells and avoid host defenses and antimicrobial agents has rendered many promising agents with direct antibacterial properties ineffective in CF. Therefore we tested the ability of cysteamine to mediate bacterial killing in human CF MDMs during a 24 hour infection by measuring total (extracellular and intracellular) and intracellular bacterial growth. Human CF MDMs demonstrated markedly decreased total and intracellular bacterial loads of *B*. *cenocepacia and B*. *multivorans* when cysteamine was present compared to untreated macrophages (Fig 2A). *P*. *aeruginosa* total, but not intracellular bacterial load was also decreased with cysteamine (Fig 2A). However, neither MRSA total or intracellular bacterial load significantly changed with cysteamine (Fig 2A). Next, we measured macrophage viability via flow cytometry to detect non-viable, apoptotic MDMs in the presence of cysteamine alone and/or *B*. *cenocepacia* infection to ensure that cysteamine was not increasing cellular toxicity. There was no significant difference in apoptosis of non-CF and CF MDMs during *B*. *cenocepacia* infection, cysteamine exposure, or *B*. *cenocepacia* and cysteamine combined (Fig 2B). Additionally, cysteamine did not increase apoptosis during *B*. *cenocepacia* infection compared to infection alone.

### Cysteamine decreases TG2 accumulation and increases CFTR expression

**Fig 3:**
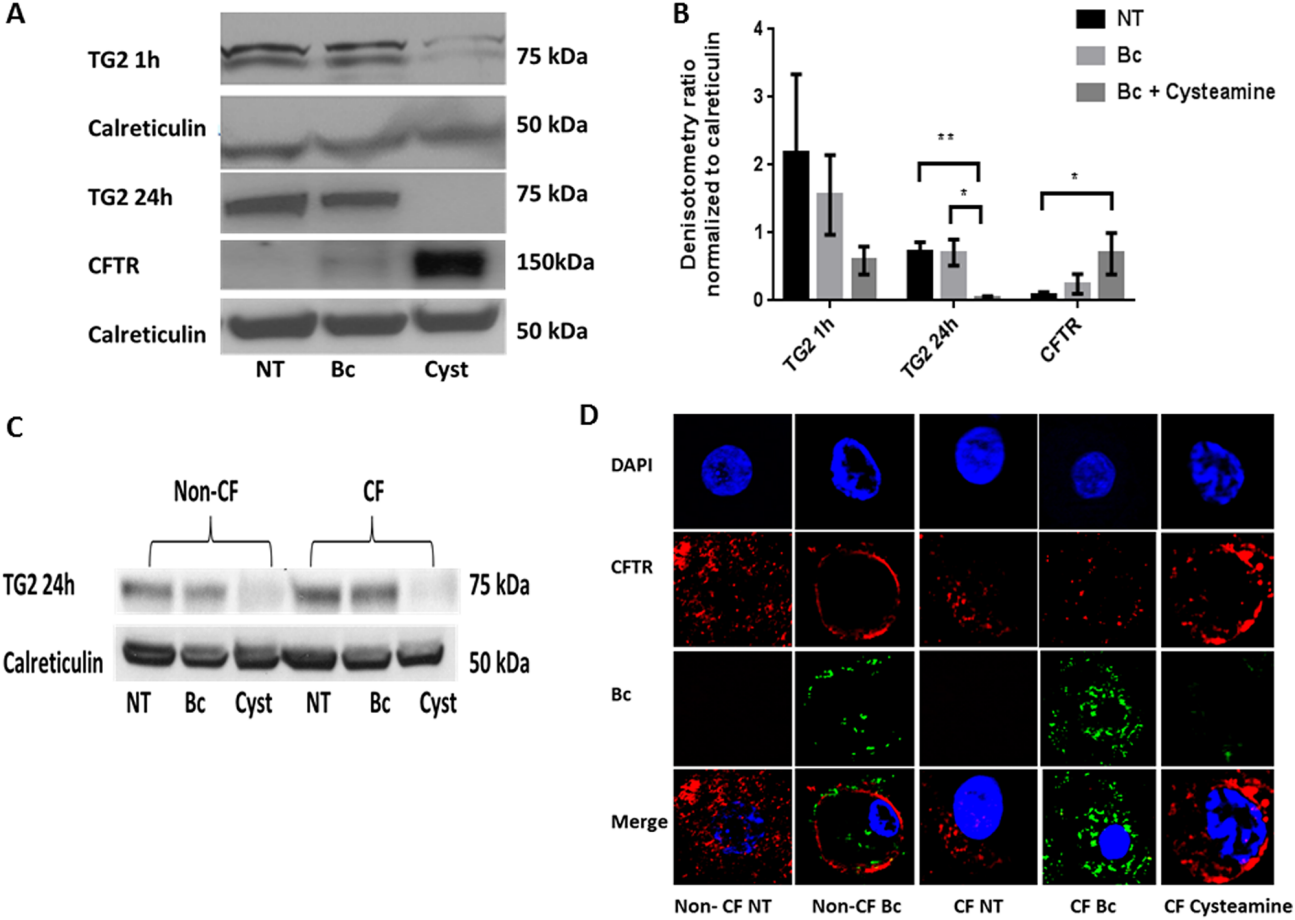
Cysteamine decreases TG2 and increases CFTR expression in CF. A) Representative Western blot of TG2 and CFTR expression in human CF MDMs at baseline (NT) and after 1 or 24h infection with *B*. *cenocepacia* (Bc) with or without cysteamine. CFTR expression is representative of both class 1 and class II mutations. B) Densitometric analysis of ≥ 3 Western blots per TG2 and CFTR conditions in 3A, normalized to the loading control calreticulin. “*” denotes a p value < 0.05, “**” denotes a p value < 0.01 for 2-sample comparisons. For CFTR expression, 3 patients with a second class I mutation and 3 with a second class II mutation are presented as aggregate data. C) Representative Western blot of TG2 expression in human non-CF and CF MDMs at baseline (NT) and after 24h infection with *B*. *cenocepacia* (Bc) with or without cysteamine (cyst). D) Confocal microscopy images of CFTR expression in non-CF and CF human MDMs at baseline (NT) and infected with *B*. *cenocepacia* (Bc). CF MDMs were additionally treated with cysteamine during Bc infection (Cysteamine). The macrophage nucleus is stained blue with DAPI, *B*. *cenocepacia* (Bc) is shown in green, and CFTR expression in red.

CF epithelial cells and CF mice demonstrate elevated TG2 levels which can be reduced with cysteamine [11]. Because cysteamine was effective in reducing CF macrophage bacterial burden, we examined TG2 expression in human CF MDMs. CF MDMs demonstrated high TG2 expression basally and with *B*. *cenocepacia* infection (Fig 3A and 3B). Cysteamine reduced TG2 expression in infected CF MDMs under both short (1h) and long (24h) exposure durations, with significant decreases observed at 24h (Fig 3A and 3B). Cysteamine also reduced TG2 expression in non-CF macrophages, which display lower expression of TG2 at baseline and during infection compared to CF. (Fig 3C). We then examined CFTR expression in CF MDMs as elevated TG2 leads to CFTR aggresomes, whereby CFTR expression is decreased due to unavailability of CFTR for trafficking to cell membranes [11]. CF MDMs from patients with at least one copy of the classic CF class II mutation F508del and a second class I (n = 3) or class II (n = 3) mutation were utilized. CF MDMs demonstrated minimal CFTR expression at baseline and during *B*. *cenocepacia* infection (Fig 3A and 3B). Cysteamine significantly increased human MDM CFTR expression (Fig 3A and 3B). Confocal microscopy was then performed to determine if CFTR expression changes correlated with functional changes. Microscopy demonstrated the CFTR expression was diminished in CF MDMs at baseline and during *B*. *cenocepacia* infection, in contrast to non-CF MDMs which demonstrated robust expression and re-organization to the peripheral membrane during *B*. *cenocepacia* infection (Fig 3D). Cysteamine treatment increased CF MDM CFTR expression and re-organization to the peripheral membrane (Fig 3D), correlating with the Western blot data.

### Cysteamine increases CF macrophage autophagy

**Fig 4:**
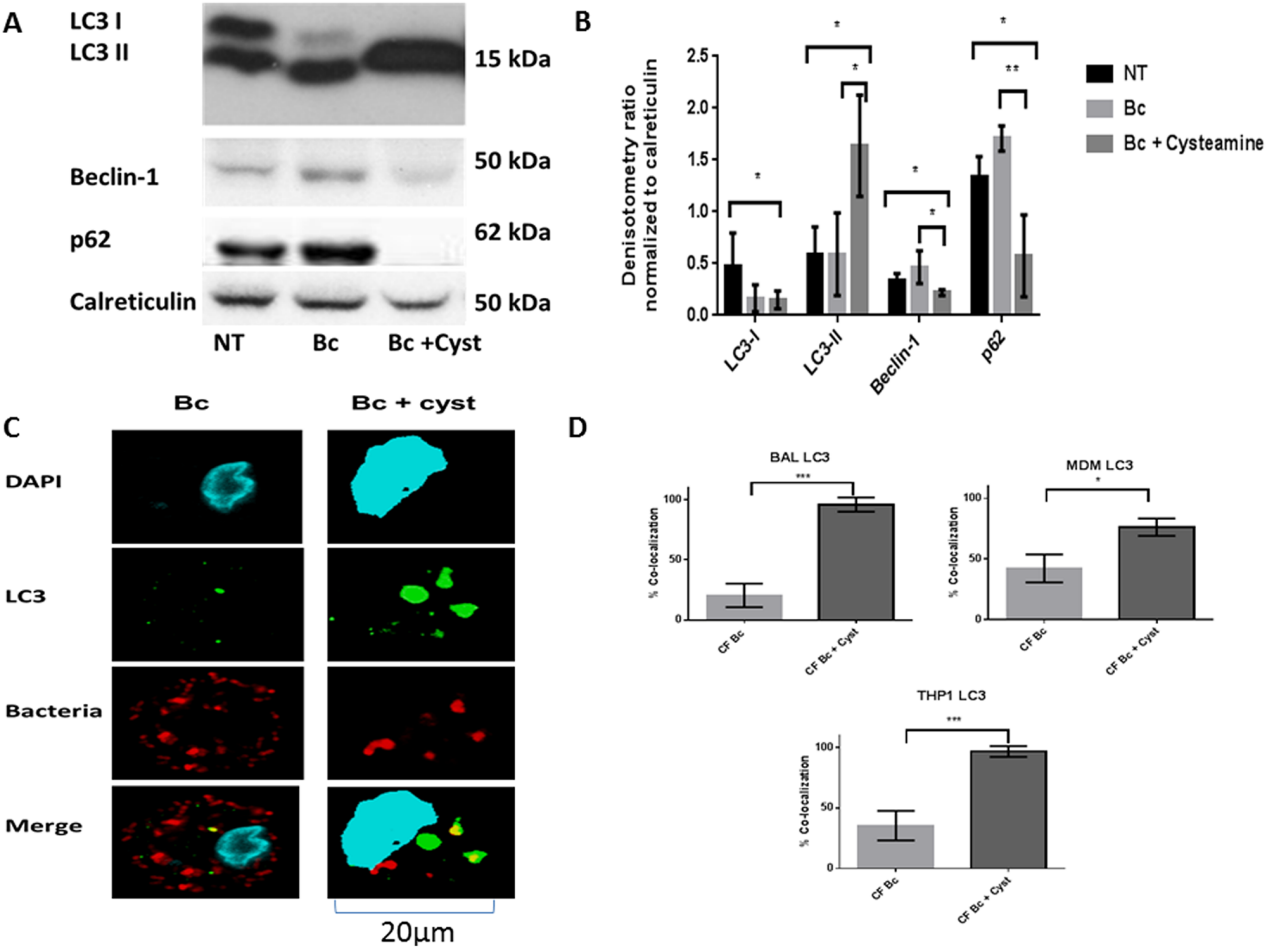
Cysteamine increases CF macrophage autophagy. A) Western blot of LC3, beclin-1, and p62 expression in human CF MDMs at baseline (NT) or following 24h infection with *B*. *cenocepacia* (Bc) with or without cysteamine. B) Densitometric analysis of ≥ 3 Western blots per condition in 4A, normalized to the loading control calreticulin. C) Confocal microscopy images of CF human alveolar macrophages infected with *B*. *cenocepacia* with or without a 24h of cysteamine and analyzed for autophagosome formation (LC3). The macrophage nucleus is stained blue with DAPI, *B*. *cenocepacia* (Bc) is shown in red, LC3 is shown in green, and bacteria co-localized with LC3 are yellow in the merged image. D) Summed scoring of bacterial co-localization from 4C (BAL, n = 1) as well as MDMs (n = 3) and THP-1 macrophages (n = 3). 100 MDMs scored per condition. BAL p value <0.0001, MDM p value = 0.02, THP-1 p value < 0.0001. “*” denotes a p value < 0.05, “**” denotes a p value < 0.01, and “***” denotes a p value < 0.001, 2-sample comparisons.

**Fig 5:**
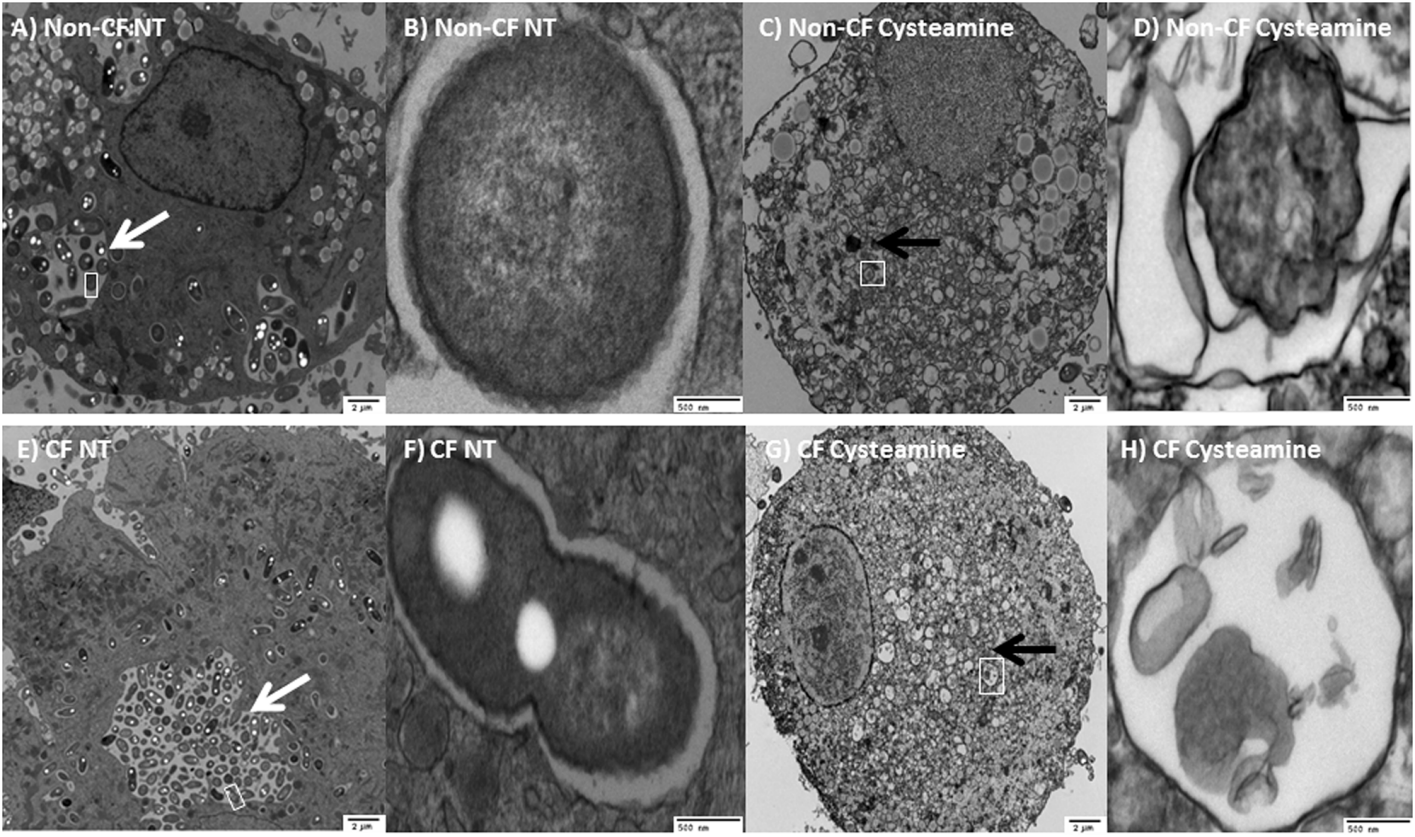
Cysteamine increases single-membrane autophagosome formation. Electron microscopy (EM) images of CF and non CF MDMs during *B*. *cenocepacia* infection with or without cysteamine. A) Untreated non-CF MDMs, magnification 4500X, 2μm. White arrow indicates vacuole with few bacteria. Square indicates zoom area for 5B. B) Untreated non-CF MDM, zoom 34000X, 500nm. C) Treated non-CF MDMs, 4500X, 2μm. Black arrow indicates clearance of bacteria. Square indicates zoom area for 5D. D) Treated non-CF MDM, zoom 34000X, 500nm. E) Untreated CF MDMs, 4500X, 2μm. White arrow indicates large vacuole of bacteria. F) Untreated CF MDM, zoom 34000X, 500nm. G) Treated CF MDMs, 4500X, 2μm. Black arrow indicates clearance of bacteria, square indicates zoom area for 5H. H) Treated CF MDM, zoom 34000X, 500nm.

CF macrophages have deficient autophagy characterized by aggregation of beclin-1and p62 with resulting decreased autophagosome formation. *B*. *cenocepacia* can further suppress autophagy in CF [2, 9, 29]. We examined expression of autophagy markers basally, during *B*. *cenocepacia* infection, and with the addition of cysteamine during infection in MDMs. Cysteamine increased conversion of LC3-I to LC3-II (indicative of autophagosome formation), decreased beclin-1 expression, and decreased p62 expression (Fig 4A and 4B). We then confirmed increased autophagosome formation via confocal microscopy using human CF MDMs, alveolar macrophages, and THP-1 derived macrophages. All CF macrophage types demonstrated increased co-localization of *B*. *cenocepacia* with LC3 after cysteamine, indicative of localization into autophagosomes (Fig 4C and 4D). In order to further characterize the nature of the autophagosomes induced by cysteamine, EM was performed in human CF MDMs during *B*. *cenocepacia* infection with and without cysteamine (Fig 5). EM images were notable for high bacterial burden including large vacuoles with replicating bacteria under low and high-power magnification in untreated CF MDMs compared to non-CF MDMs (Fig 5A, 5B, 5E and 5F). Cysteamine significantly reduced bacteria in both CF and non-CF MDMs with high-power magnification demonstrating individual vacuoles with degraded bacterial contents (Fig 5C, 5D, 5G and 5H).

**Fig 6:**
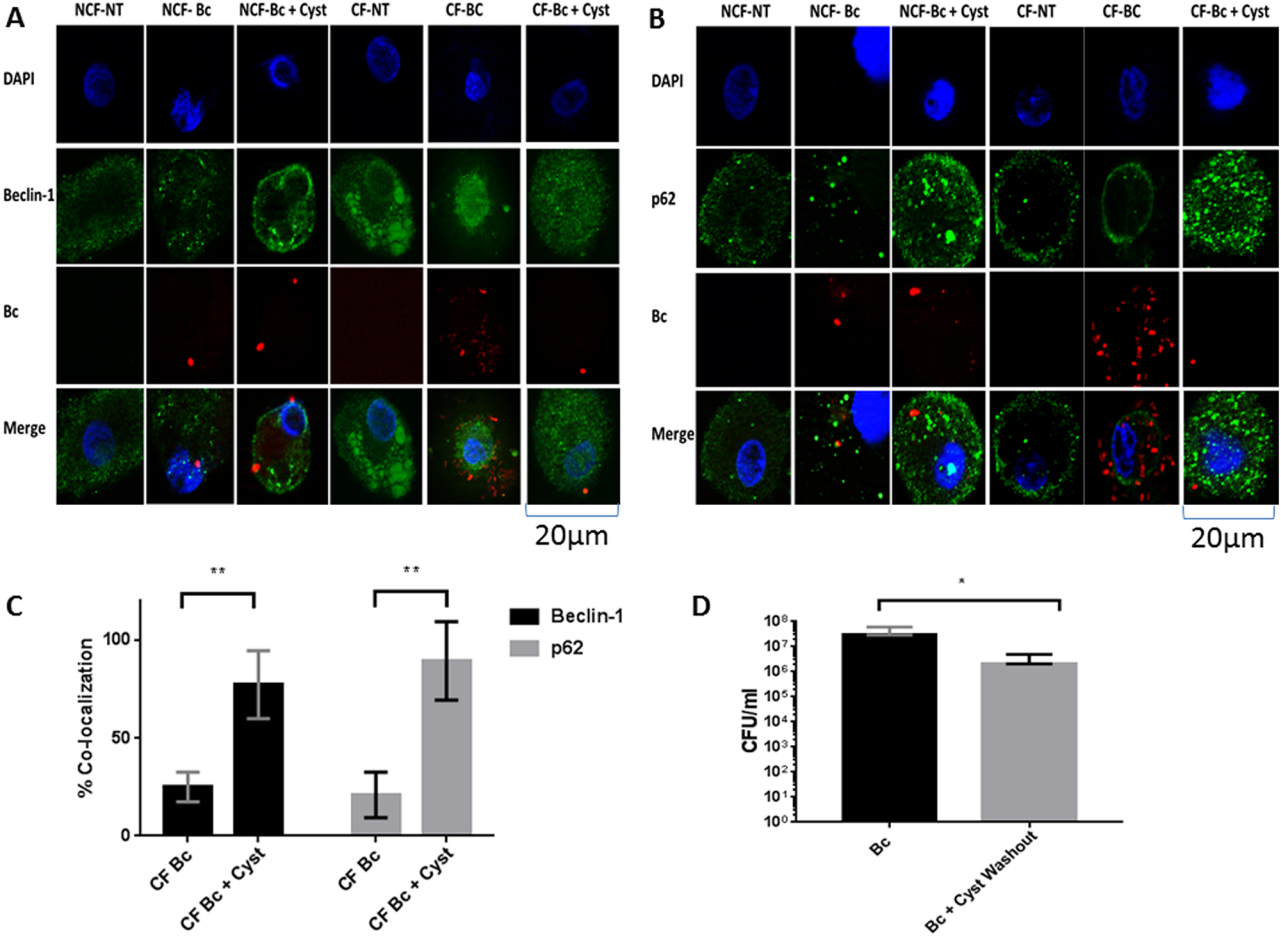
Cysteamine regulates autophagy proteostasis. Confocal microscopy images of CF MDMs infected *B*. *cenocepacia* with or without a 24h of cysteamine and analyzed for (A) beclin-1 and (B) p62. The macrophage nucleus is stained blue with DAPI, *B*. *cenocepacia* (Bc) is shown in red, beclin-1 and p62 are shown in green in their respective sections, and bacteria co-localized with beclin-1 or p62 are yellow in the merged image. C) Summed scoring of bacterial co-localization from 6A and 6B. 100 MDMs scored per condition. “**” denotes a p value < 0.01. D) Colony forming unit (CFU) assay for human CF MDMs infected with *B*. *cenocepacia* (Bc) for 24h with or without cysteamine, n = 4. Cysteamine was removed for 24h prior to infection (washout). Intracellular (IC) only counts are displayed. “*” denotes a p value < 0.05.

To verify decreased beclin-1 and p62 expression, confocal microscopy was performed. Microscopy demonstrated that beclin-1 was aggregated into large clumps at baseline (CF beclin-1 mean area 23.8 ± 53.8 pixels vs non-CF 1.5 ± 0.7 pixels) and during *B*. *cenocepacia* infection (CF beclin-1 mean area 157.1 ± 916.1 pixels vs non-CF 14.1 ± 25.4 pixels) in CF compared to non-CF MDMs (Fig 6A). CF MDM beclin-1 aggregation decreased with cysteamine (CF beclin-1 mean area 13.0 ± 34.2 pixels). p62 was noted to be in a peripheral distribution in CF MDMs basally without clumping. CF MDM p62 demonstrated a similar distribution pattern throughout the cell to non-CF MDMs after cysteamine (Fig 6B). CF MDMs demonstrated decreased co-localization of beclin-1 and p62 with *B*. *cenocepacia* (Fig 6C). Upon cysteamine addition, co-localization with bacteria increased in CF MDMs coincident with decreased bacteria counts (Fig 6A, 6B and 6C). To confirm the finding that decreased bacterial load was dependent on cysteamine induction of macrophage autophagy and not direct bacterial killing, bacterial CFUs were measured in CF MDMs exposed to cysteamine with washout of cysteamine prior to infection with *B*. *cenocepacia*. Cysteamine-exposed CF MDMs demonstrated a 2 log reduction in bacterial growth (Fig 6D), consistent with prior results.

### Cysteamine decreases ROS and inflammatory cytokine production

**Fig 7:**
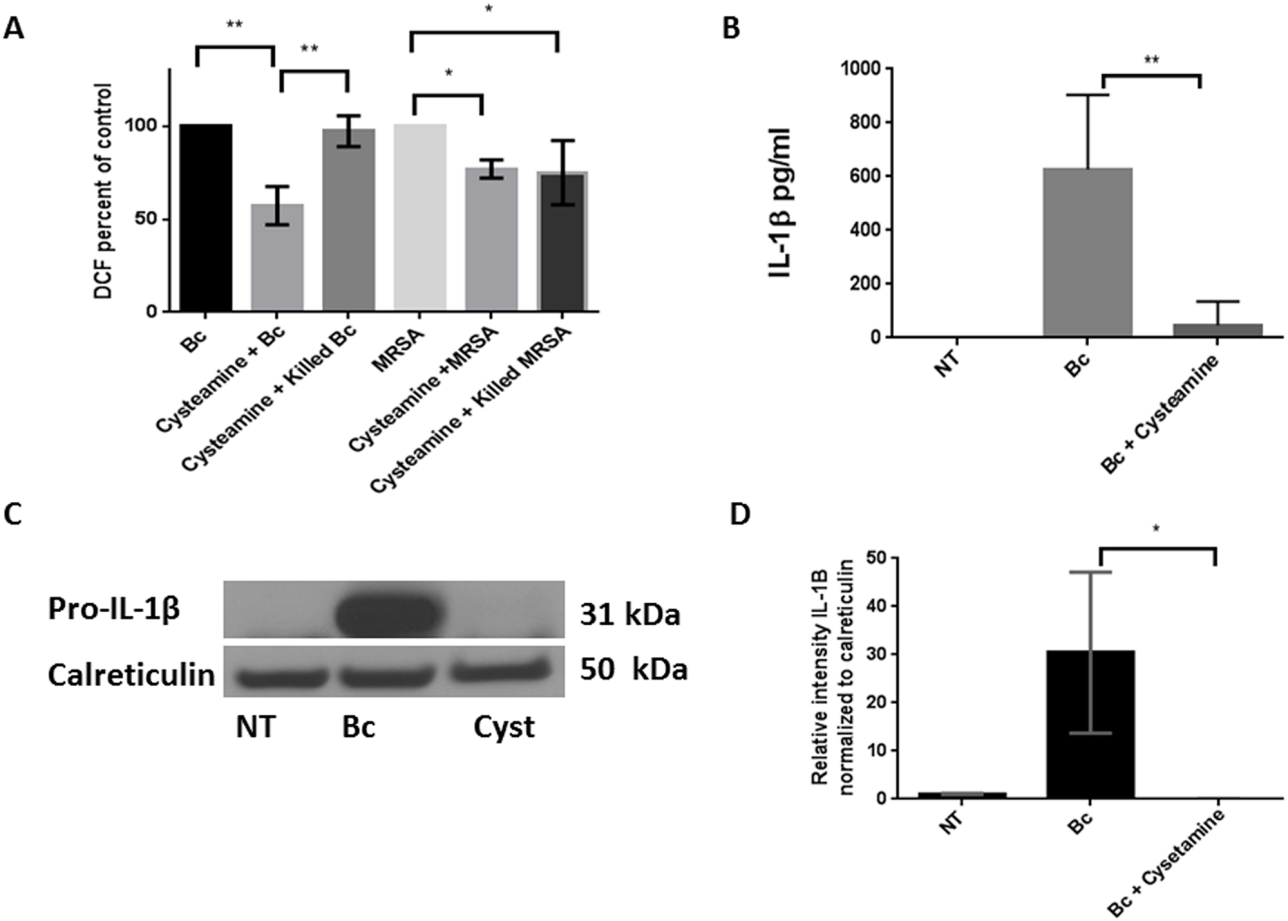
Cysteamine reduces ROS and inflammatory cytokine production. A) Summed end-point analysis expressed as %ROS production at 2h of CF MDMs infected with live or paraformaldehyde-killed *B*. *cenocepacia* or MRSA with or without cysteamine and assessed for ROS production using RFUs via a DCF assay. Results normalized to live *B*. *cenocepacia* and live MRSA. Representative assay of n = 3, “*” denotes a p value < 0.05, “**” denotes a p value < 0.01. B) IL-1β ELISA for CF MDM supernatants basally (NT), or following 24h *B*. *cenocepacia* infection (Bc), or Bc infection plus cysteamine, n = 4, p value = 0.007. C) Western blot of pro-IL-1β expression in human CF MDM lysates basally (NT) or following 24h infection with *B*. *cenocepacia* (Bc), with or without cysteamine. D) Densitometric analysis of ≥ 3 Western blots per condition in 7D, normalized to the loading control calreticulin. “*” denotes a p value < 0.05, “**” denotes a p value < 0.01, and “***” denotes a p value < 0.001.

Constitutive ROS production in CF airways promotes inflammation and drives TG2-mediated cross-linking and sequestration of autophagy proteins. We therefore examined ROS production in response to the first 2h of *B*. *cenocepacia* infection when exposed to cysteamine. Cysteamine significantly decreased ROS production in CF MDMs by 48.7% during infection (Fig 7A, p value < 0.001). However, there was no change in ROS production in response to paraformaldehyde-killed *B*. *cenocepacia* (Fig 7A). ROS production in response to MRSA was also examined as a control since cysteamine did not decrease MRSA CFUs. Cysteamine reduced ROS by 23% in response to both live and PFA-killed MRSA (Fig 7A). Taken together, these results suggest an effect of cysteamine on ROS production independent of bacterial burden.

In addition to ROS production, *B*. *cenocepacia* infections in CF are characterized by exaggerated cytokine production such as pro-inflammatory IL-1β, which can lead to prolonged inflammation and further ROS release [14]. Cysteamine abolished IL-1β production in CF MDM cell supernatants during *B*. *cenocepacia* infection (Fig 7B). Decreased IL-1β production was confirmed by Western blotting of cell lysates which demonstrated decreased pro-IL-1β expression during cysteamine exposure (Fig 7C and 7D).

## Discussion

Chronic bacterial infections remain a consistent problem for patients with CF despite advances in CF treatment including CFTR modulators. Deficits in CF host immune responses have combined with increased anti-microbial resistance to lead to a heightened need for improved understanding of host-pathogen interactions in CF. To this end we have demonstrated that cysteamine is able to improve human macrophage-mediated killing of pathogens such as *B*. *cenocepacia* that are otherwise recalcitrant to standard therapeutic regimens.

To date, we lack a gold standard treatment regimen for *B*. *cenocepacia* and other multi-drug resistant organisms in CF. Survival of *B*. *cenocepacia* in patients with CF is predicated upon the ability of the bacteria to live intracellularly in macrophages and avoid normal host defenses including autophagy [2, 9]. Development of anti-microbial compounds that show promise against *B*. *cenocepacia* directly often lack therapeutic efficacy if host macrophages are not targeted. Cysteamine demonstrated both an ability to directly kill *B*. *cenocepacia*, as well as enhance host-mediated killing via increased autophagy. These results suggest that cysteamine is a promising agent alone, or in combination with antibiotics for the treatment of *B*. *cenocepacia* and other resistant organisms in CF. Our results support murine studies utilizing cysteamine during *P*. *aeruginosa* infection [21], but the lack of intracellular *P*. *aeruginosa* killing may reflect the need for other host cell involvement such as neutrophils. Cysteamine did not reduce MRSA CF macrophage burden, despite direct growth inhibition of MRSA in the absence of macrophages. We speculate that this may be due to either increased MRSA resistance to autophagy or oxidative killing, or potentially other MRSA-associated virulence mechanisms. *Staphylococcus aureus* has been previously shown to require autophagy for continued replication [30], while other groups have demonstrated that it can alter autophagic flux to provide a survival niche within autophagosomes. [31] Additionally, *S*. *aureus* interactions with the autophagy pathway may proceed through a non-canonical path regulated by intracellular levels of cAMP [32]. Together, these studies support a role for MRSA regulation through specialized autophagy pathways to help explain the persistence of MRSA in CF macrophages exposed to cysteamine, but further studies in CF are needed.

In addition to decreasing bacterial load, cysteamine was able to reduce continued ROS and inflammatory cytokine production. *B*. *cenocepacia* infections in CF are characterized by exaggerated pro-inflammatory IL-1β production [14], which cysteamine was able to markedly reduce. These findings suggest that cysteamine use can reduce systemic inflammation caused by *B*. *cenocepacia* infection, likely in part due to reduced bacterial burden. Reduction of cytokine and oxidant-induced stress also reduces signals that inhibit functional autophagy, such as TG2 production. Therefore, cysteamine is able to reduce sequelae of the pathogen and improve overall host defense.

Cysteamine was able to increase CFTR expression in macrophages from patients with class I and class II classical CF mutations. Therefore, we would anticipate broad applicability across severe mutation classes in regards to cysteamine’s impact on increasing macrophage CFTR expression when at least one copy of the F508del mutation is present. Other classes of CFTR expression were not available for this study. Increased CFTR expression was also coincident with re-arrangement of CFTR, suggestive of increased CFTR trafficking and function. Our work supports prior studies using cysteamine in class II CF mutations [33].

Despite its many benefits, cysteamine is associated with several gastrointestinal (GI) side effects including abdominal bloating and nausea [22, 34]. We observed that cysteamine killed several gram-negative bacteria that are either resident or pathogenic to the GI tract. We hypothesize that disruption of the gut microbiome during cysteamine exposure is a potential mechanism of GI-related side effects during systemic cysteamine use. Cysteamine is also used to induce GI ulcers in experimental models and may impact acid secretion as other potential mechanisms of GI side effects [35]. The potential use of probiotic supplements (such as containing *L*. *reuteri)* to maintain a healthy GI microbiota during systemic cysteamine treatment would need consideration of our results which demonstrated bacterial load reduction during cysteamine treatment. For patients with CF, GI side effects could be potentially mitigated with aerosol or nano-based delivery of cysteamine to minimize an impact on the GI microbiome [36].

In summary, herein we demonstrate that cysteamine is able to clear *B*. *cenocepacia* and other pathogens from CF macrophages by inhibiting TG2 thereby reducing the accumulation of p62, restoring beclin-1, and thus re-establishing autophagy in CF macrophages. Cysteamine also has potent direct growth inhibition effects on multiple pathogens. Thus, cysteamine may be an effective adjunct to antibiotic therapies in CF.
